# The oncogenic role of hypomethylated *ZNF793* in gastric carcinoma: a focus on cell survival and stemness

**DOI:** 10.1007/s10120-025-01632-8

**Published:** 2025-06-22

**Authors:** Lingyan Jin, Hye-Yeong Jin, Meihui Li, Younghoon Kim, Nam-Yun Cho, Jeong Mo Bae, Gyeong Hoon Kang

**Affiliations:** 1 Laboratory of Epigenetics, Cancer Research Institute, Seoul National University College of Medicine, Seoul, South Korea; 2 Department of Hospital Pathology, Seoul St. Mary’s Hospital, College of Medicine, The Catholic University of Korea, Seoul, Korea; 3 Department of Pathology, Seoul National University College of Medicine, 103 Daehak-Ro, Chongno-Gu, Seoul, 03080 South Korea

**Keywords:** EBV, Gastric carcinoma, Methylation, Tumor stemness, ZNF793

## Abstract

**Background:**

Although genome-wide promoter CpG island hypermethylation of Epstein–Barr virus-associated gastric carcinoma (EBV GC) is well known, *ZNF793* is rarely methylated in EBV GC but is frequently methylated in other molecular subtypes of GC, including microsatellite instability-high GC. Based on the hypothesis that ZNF793 may be important for cell survival and stemness in EBV GC, the oncogenic role of ZNF793 was investigated.

**Methods:**

ZNF793 expression was knocked out and then restored in EBV and non-EBV GC cell lines, and its effects on cell proliferation, cell migration, cell invasion, tumor sphere formation, and xenograft tumor formation were assessed.

**Results:**

ZNF793 knockout significantly suppressed the migration, invasion, proliferation, and stemness in GC cells with ZNF793 expression. Additionally, ZNF793 knockout significantly inhibited tumor growth in a xenograft tumor model.

**Conclusions:**

These results suggest that ZNF793 plays an oncogenic role in not only EBV GC but also other subtypes of GC and may be a potential therapeutic target.

**Supplementary Information:**

The online version contains supplementary material available at 10.1007/s10120-025-01632-8.

## Introduction

Gastric carcinoma (GC) is a common and deadly cancer worldwide, ranking fifth in both incidence and mortality according to global cancer statistics for the year 2022 [1]. GC patients have different survival rates depending on the molecular subtype; GC subtypes include Epstein‒Barr virus (EBV), microsatellite instability (MSI), genomic stability, and chromosomal instability [2]. The development of therapies tailored to the molecular subtype is essential to improving the survival rate of GC patients. The EBV subtype accounts for approximately 10% of GCs [3] and is characterized by extensive genome-wide promoter CpG island hypermethylation; it has the highest rate of promoter DNA hypermethylation among various human cancer tissue types [2]. Along with the MSI subtype, the EBV subtype constitutes the CpG island methylator phenotype (CIMP), which is characterized by concurrent genome-wide promoter CpG island hypermethylation [2, 4–6]. However, the number of methylated CpG island loci is greater in EBV GC than in MSI GC, and the vast majority of the genes that are methylated in MSI GC are also methylated in EBV GC [2, 6]. Nevertheless, of the genes whose promoter CpG island loci and expression are methylated and suppressed, respectively, in MSI GC, a small subset of genes, including *MLH1*, are unmethylated and expressed in EBV GC [7].

Zinc finger (ZNF) proteins, which constitute the largest family of transcription factors in the human genome, play a role in initiating and promoting the malignant progression of different cancers, including GC [8]. ZNF793 is a member of the Krüppel-associated box (KRAB)-containing zinc finger protein (KZFP) family and has a KRAB domain and a tandem array of C2H2 zinc fingers at the N- and C-termini, respectively [9]. In the human genome, more than 380 genes encode KZFPs [10], many of which work with KAP1 to silence retrotransposon expression through epigenetic modification by recruiting the histone methyltransferase SETDB1 and the nucleosome-remodeling and histone deacetylation complex [9, 11]. Some KZFPs have also been demonstrated to act as oncoproteins in cancer cells, promoting cell growth and invasiveness, and to play a key role in preserving stemness [12]. Although ZNF793 has been shown to interact with KAP1 and SIRT1 [13], the biological function of ZNF793 remains unclear. 

In a previous study [14], we found that not only *MLH1* but also *ZNF793* is highly methylated in MSI GCs, resulting in repression of ZNF793 expression, whereas *ZNF793* is unmethylated and expressed in EBV GCs (Supplementary Fig. 1a and b). The question of why EBV GCs, despite their genome-wide hypermethylation, maintain the unmethylated status of *ZNF793*, which is commonly methylated in GCs of other molecular types (Supplementary Fig. 1b), led us to hypothesize that ZNF793 expression may play an important role in maintaining cell survival or stemness in a subset of GCs. To test this hypothesis, we knocked out *ZNF793* in EBV and non-EBV GC cell lines and then investigated whether ZNF793 plays an oncogenic role, especially in the context of stemness.

## Materials and methods

### Tissue samples and DNA extraction

The Institutional Review Board of Seoul National University Hospital approved the study protocol (H-2412–139-1600). The study was conducted in accordance with the Declaration of Helsinki. From 483 patients with advanced GC who underwent surgery at Seoul National University Hospital between 2007 and 2008, archival tissue blocks with high tumor cellularity and representative histology were chosen by reviewing histological slides. MSI and EBV status data for this GC cohort were obtained from previous studies [15–17]. DNA was extracted from unstained tissue slides by scraping the tumor tissues into microtubes containing lysis buffer and proteinase K.

### Bisulfite modification and the MethyLight assay

The genomic DNA was bisulfite modified using the EZ DNA Methylation Kit (Zymo Research, Irvine, CA, USA). The bisulfite-modified DNA was subjected to the MethyLight assay as previously described [18], and the percentage of the methylated ratio was determined for the ZNF793 promoter CpG island locus. The oligonucleotide sequences of the primers and probe are shown in Supplementary Table 1**.**

### Cell culture

The non-EBV GC cell line (AGS, SNU1, SNU5, SNU620, and SNU638) and EBV GC cell lines (SNU719 and NCC24) were purchased from the Korean Cell Line Bank (Seoul, Korea). Another EBV GC cell line, YCCEL1, was kindly provided by Dr. Sun-Young Rha at the Songdang Institute for Cancer Research, Yonsei University College of Medicine (Seoul, Korea). All the cells were cultured in RPMI 1640 (Welgene, Gyeongsan-si, Gyeongsangbuk-do, Korea), which contained 10% FBS (Gibco, New York, USA) and 1% penicillin ‒ streptomycin solution (Welgene). The e-Myco™ plus Mycoplasma PCR Detection Kit (ver 2.0) (iNtRON Biotechnology, Seongnam-si, Gyeonggi-do, Korea) was used to ensure that all cell cultures were free of mycoplasma contamination.

### Plasmid transfection and lentiviral transduction

Single-guide RNAs (sgRNAs) that target *ZNF793* or the control were inserted into the LentiCRISPRv2 vector (#52,961, Addgene Inc., Cambridge, MA, USA) and used to transfect HEK293FT cells with ViraPower™ Packaging Mix (Invitrogen, Carlsbad, CA, USA) and Lipofectamine 2000 Transfection Reagent (Thermo Fisher Scientific, Waltham, MA, USA) to produce a lentivirus-containing supernatant. AGS and SNU719 cells were transduced with the lentiviruses for 48 h with 10 μg/ml polybrene (Merck, Darmstadt, Hesse, Germany). The transduced cells were then treated with 2 μg/ml puromycin (Sigma‒Aldrich, St. Louis, MO, USA) for 7 days for selection. Control and ZNF793 knockout cells were subsequently plated in 96-well plates as single cells in medium supplemented with puromycin for culture. The relevant sequences are shown in Supplementary Table 2.

The ZNF793 expression plasmid (ZNF793 (NM 001013659) Human Untagged Clone, Origene Technologies, Rockville, MD, USA) and the control plasmid (pCMV6-Entry, Origene Technologies) were utilized to induce the expression of ZNF793 in AGS and SNU719 cells. The transfection was conducted using Lipofectamine 2000 with an incubation period of 72 h. Then, the transfected cells were selected with G418 solution (Sigma‒Aldrich) at a concentration of 2 mg/ml for 7 days.

### siRNA transfection

ZNF793 siRNA (h) (sc-97736, Santa Cruz Biotechnology, Inc., Dallas, TX, USA) and control siRNA (Fluorescein Conjugate)-A (sc-36869, Santa Cruz Biotechnology, Inc.) were used for the downregulation of ZNF793 in SNU620 cell. Transfection was conducted using Lipofectamine 2000 with an incubation period of 48 h.

### RNA extraction and real-time qPCR

Following the manufacturer's instructions, we extracted RNA with the RNeasy Plus Mini Kit (Qiagen, Hilden, Germany) and synthesized cDNA with RevertAid Master Mix (Thermo Fisher Scientific). Real-time polymerase chain reaction (qPCR) was performed using Power SYBR Green PCR Master Mix (Applied Biosystems, Foster City, CA, USA) and HotStarTaq Plus DNA Polymerase (Qiagen) on a CFX384 Touch Real-Time PCR System (Bio-Rad, Hercules, CA, USA). GAPDH was used as an internal control, and relative expression was analyzed via the 2^−ΔΔCT^ method. The sequences of primers used for RT‒qPCR are shown in Supplementary Table 3.

### Western blotting

Cells and tissues were lysed in RIPA buffer (Merck, Darmstadt, Germany) supplemented with protease inhibitor (Roche, Mannheim, Germany) and phosphatase inhibitor cocktail (Roche) to extract total protein. The Pierce™ BCA Protein Assay Kit (Thermo Fisher Scientific) was used to measure the protein concentration. Proteins were quantified and run on 12% SDS‒polyacrylamide gels and then transferred to PVDF membranes (Merck Millipore, Darmstadt, Germany) by electrophoresis. The membranes were blocked with 5% skim milk (BD Difco, Franklin Lakes, NJ, USA) for 2 h at room temperature and then incubated overnight at 4 °C with the primary antibody. The primary antibodies used are described in Supplementary Table 4. After that, the membranes were incubated for 2 h at room temperature with diluted goat anti-mouse IgG (H + L)-HRP (GenDEPOT, Katy, Texas, USA) or mouse anti-rabbit IgG-HRP (Santa Cruz, Dallas, Texas, USA) as secondary antibodies. Clarity Western ECL Blotting Substrates (Bio-Rad) and a ChemiDoc Imaging System (Bio-Rad) were used to detect and image the protein bands. The intensities of the Western blot bands were quantified via ImageJ software and normalized to those of β-actin.

### MTT cell proliferation assay

The 96-well plates were seeded with 2.5 × 10^3^ cells per well and cultured for 1‒6 days. Each well was treated with 0.5 mg/ml MTT stock solution (10 μl of 5 mg/ml + 90 μl of RPMI 1640) and incubated for 4 h at 37 °C. The formazan that formed was dissolved in 150 μl of DMSO (Sigma‒Aldrich), and the absorbance was measured at 490 nm with a Varioskan LUX Multimode Microplate Reader (Thermo Fisher).

### Cell migration and invasion assays

The migration assay involved adding 2 × 10^4^ cells (AGS) or 5 × 10^4^ cells (SNU719) to each 8.0 μm diameter pore cell culture insert (Corning, New York, USA) and incubating them for 48 (AGS) or 96 (SNU719) hours in a cell culture incubator. The non-invasive cells were then gently removed with wet cotton-tipped swabs. The migrated cells were stained with crystal violet.

For the invasion assay, the Matrigel matrix (Corning) was diluted with PBS to 300 μg/ml, and each insert was coated with 0.2 ml of the mixture and then incubated at 37 °C for 2 h. The PBS was removed from the inserts without touching the basement membrane layer. The inside of each insert was seeded with 2 × 10^4^ cells (AGS) or 5 × 10^4^ cells (SNU719), and the lower well of the 24-well plate was filled with 600 μl of media containing 10% FBS. After culturing for 48 (AGS) or 96 (SNU719) hours in a cell culture incubator, noninvasive cells were gently removed using wet cotton-tipped swabs. Invaded cells were stained with crystal violet.

### Apoptosis assay

Apoptosis was analyzed via Annexin V labeling using an Annexin V-FITC detection kit (Invitrogen, California, USA). Briefly, the cells were resuspended in binding buffer at a density of 3 × 10^6^/ml. The cell suspension was incubated with Alexa Fluor™ 488 Annexin V and propidium iodide for 15 min at room temperature in the dark and analyzed on a BD FACS Canto™ Clinical Flow Cytometry System (Biosciences, San Jose, CA, USA).

### Tumor sphere formation assay

A 6-well plate with a low-attachment surface was seeded with 2 × 10^4^ cells in 3 ml of medium without FBS (RPMI-1640, 20 ng/ml EGF, 10 ng/ml basic fibroblast growth factor, and penicillin‒streptomycin). Each well was replenished with 500 μl of medium every week, and the cells were counted after 2–3 weeks.

### Xenograft tumor growth assay

sgZNF793 or control cells were combined with Matrigel at a 1:1 ratio, and the final concentration of cells was 4 × 10^7^/ml. Then, 4 × 10^6^ cells per site were injected under the skin of BALB/c nude mice. The tumor size was periodically monitored until tumor removal. The animal experiments were approved by the Biomedical Research Institute of Seoul National University Hospital (approval no. 3520220102).

### Statistical analysis

Statistical analyses were performed via GraphPad Prism version 10 (GraphPad Software, San Diego, CA). The data were first subjected to a normality test. Statistical significance was assessed via either Student’s *t* test or the Mann‒Whitney test. All the results are shown as the means ± SDs, and P < 0.05 indicates statistical significance.

## Results

### Comparison of DNA methylation and ZNF793 mRNA expression between molecular subtypes of GC

**Fig. 1:**
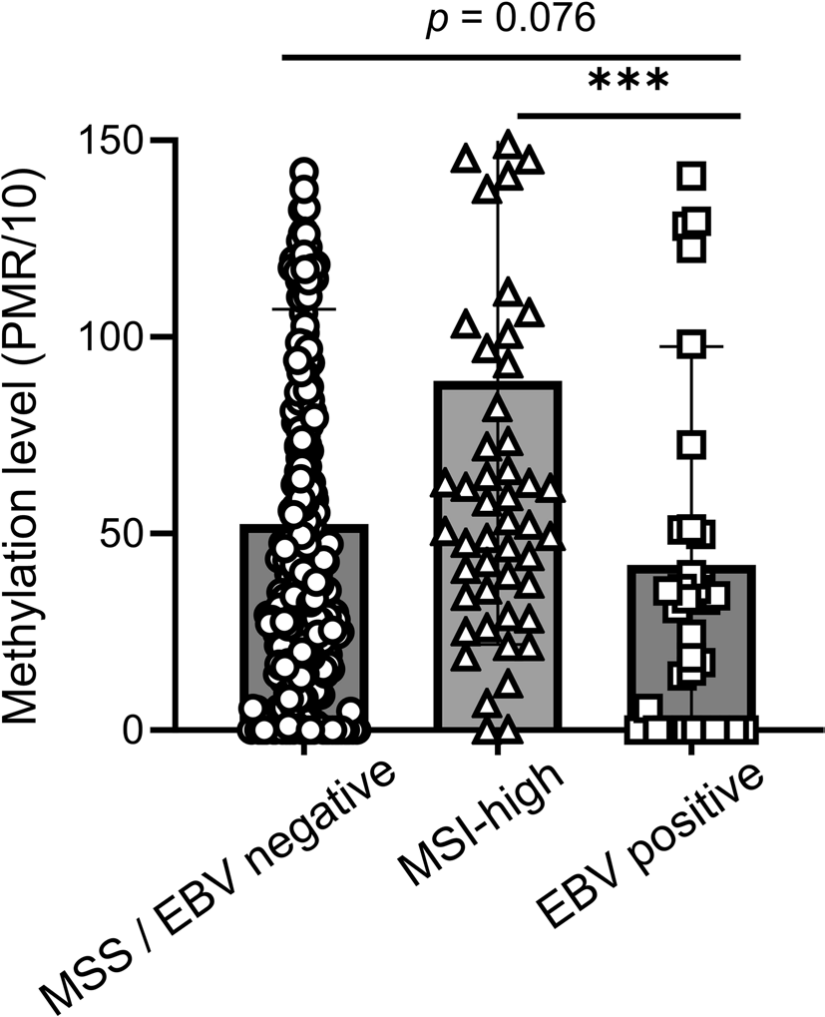
MethyLight assay results showing the methylation levels of *ZNF793* CpG island loci. The EBV subtype (n = 36) has the lowest methylation level, whereas the MSI subtype (n = 59) has the highest methylation level. The methylation level of *ZNF793* tended to be higher in microsatellite-stable (MSS)/EBV-negative (n = 394) subtype than in the EBV GC (n = 36) subtype. ***P < 0.001

Using the TCGA database, we determined the DNA methylation level of the ZNF793 CpG island locus in different molecular subtypes of GC and found that DNA methylation level was markedly lower in the EBV subtype than in other molecular subtypes of GC (Supplementary Fig. 1b). ZNF793 mRNA expression levels were notably higher in the EBV subtype than in the other molecular subtypes of GC (Supplementary Fig. 1c). To further validate this relationship, we performed correlation analysis between ZNF793 DNA methylation and mRNA expression levels across GC samples (n = 222). A significant inverse correlation was observed, indicating that hypermethylation of *ZNF793* is associated with transcriptional silencing (Supplementary Fig. 2). A detailed map was created to identify the methylation status of CpGs, including those around the transcription start site, within the ZNF793 gene and its promoter region (Supplementary Fig. 3). We also performed the MethyLight assay for *ZNF793* on DNA samples from the Seoul National University Hospital GC cohort and found that *ZNF793* methylation levels were significantly lower in EBV GC than in other molecular subtypes of GC (Fig. 1).

### ZNF793 knockout affects the migration, invasion, proliferation, and apoptosis of ZNF793-expressing cell lines

**Fig. 2:**
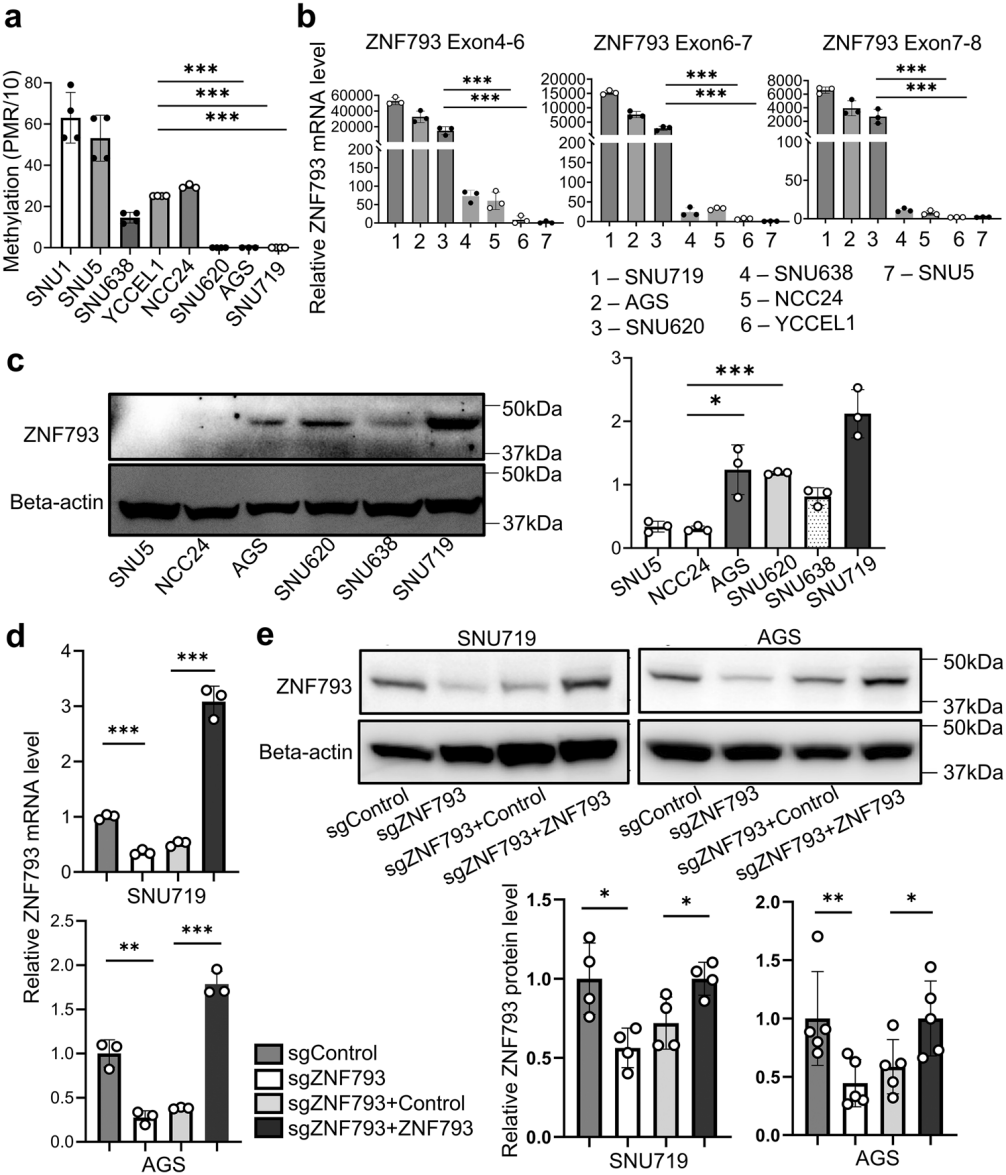
**a** Methylation levels of *ZNF793* CpG island loci in gastric cancer cell lines determined via the MethyLight assay. **b** Reverse transcription quantitative polymerase chain reaction (RT‒qPCR) probing three different regions of ZNF793 mRNA and **c** Western blotting of ZNF793 in two EBV GC cell lines (SNU719 and NCC24) and three non-EBV GC cell lines (SNU1, AGS and SNU638). **d** RT-qPCR and **e** Western blot analysis of ZNF793 in SNU719 and AGC cells with *ZNF793* knockout and *ZNF793* rescue

**Fig. 3:**
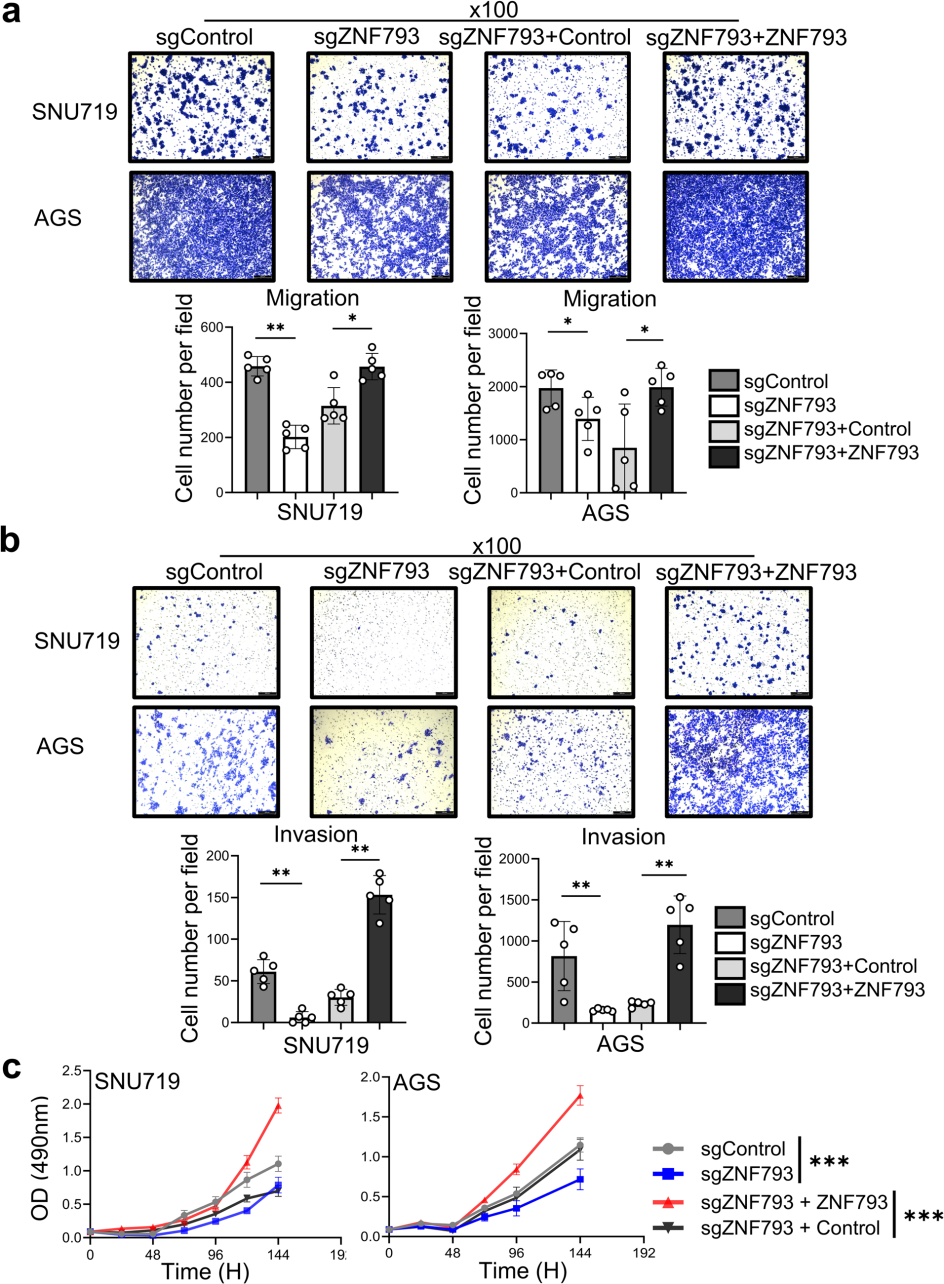
**a** Transwell migration assay (scale bar: 200 μm), **b** invasion assay (scale bar: 200 μm), **c** MTT cell proliferation assay. Cells (SNU719 and AGS) transduced with sgNC, sgZNF793, sgZNF793 plus the control vector, or sgZNF793 plus the ZNF793 expression vector

**Fig. 4:**
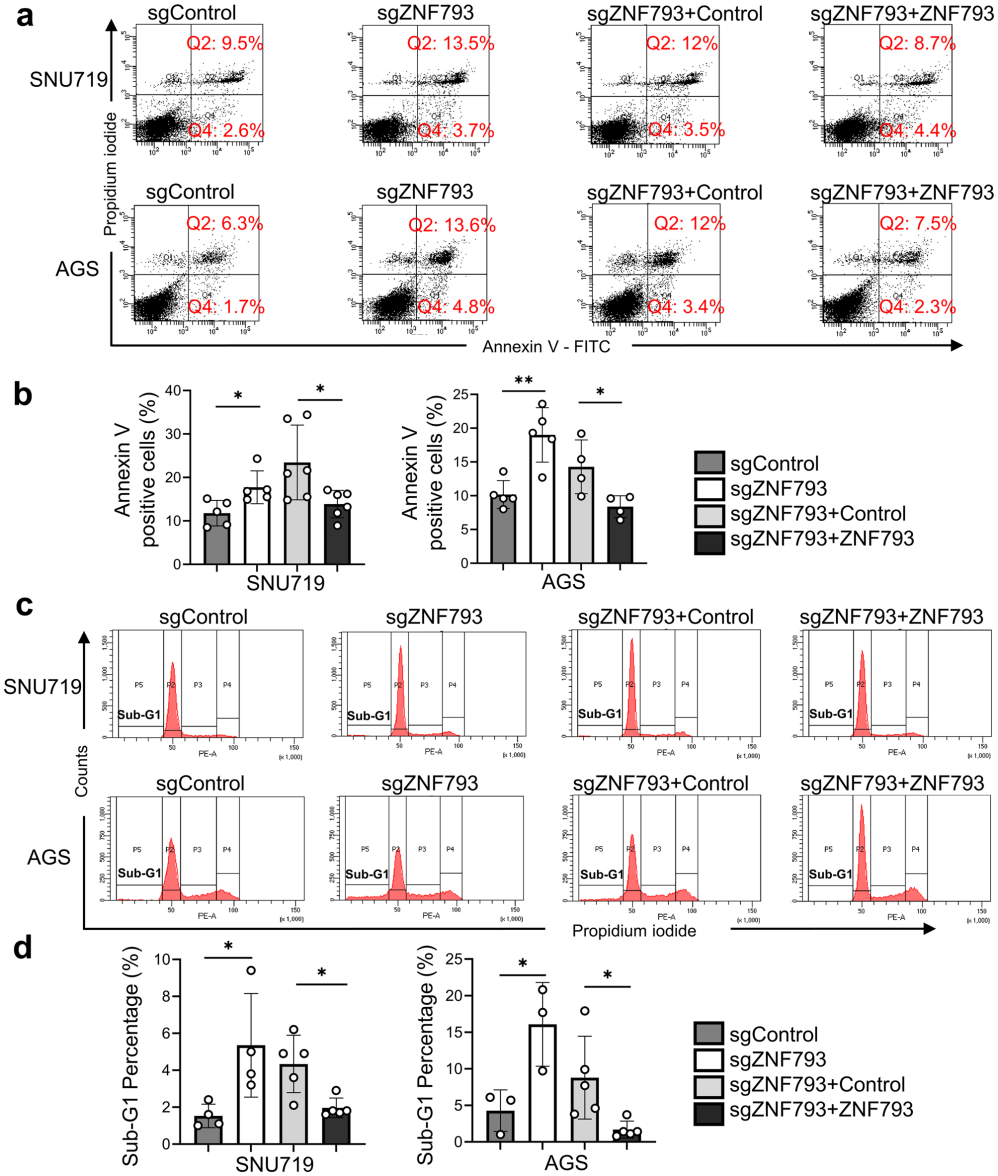
Analysis of apoptosis and the cell cycle by flow cytometry in cells (SNU719 and AGS) transduced with sgNC, sgZNF793, or sgZNF793 plus the control vector or with sgZNF793 plus the ZNF793 expression vector. **a** Representative flow cytometry plots using Annexin V-FITC/PI staining for apoptosis. SNU719 or AGS cells were treated for 48 h and then stained with Annexin V-FITC/PI for flow cytometric analysis. The squares include the following cell types: the bottom left: live cells, the bottom right: early apoptotic cells, the top right: late apoptotic cells and the top left: necrotic cells. FITC, fluorescein isothiocyanate; PI, propidium iodide. **b** The percentage of apoptotic cells (Q2 + Q4) was statistically compared among the following groups: sgNC, sgZNF793, sgZNF793 plus the control vector, and sgZNF793 plus the ZNF793 expression vector. The experiments were performed in triplicate, and the data are expressed as the mean ± standard error of the mean (SEM). **c** Cell cycle distribution was evaluated by flow cytometry. **d** The graph indicates the statistical comparison of the sub-G1 fraction between cells. *, P < 0.05; **, P < 0.01

Three EBV GC cell lines were assessed, and *ZNF793* was found to be methylated and inactive in NCC24 and YCCEL1 cells but unmethylated and active in SNU719 cells (Fig. 2a–c). *ZNF793* was also unmethylated and expressed in the non-EBV GC cell lines, AGS and SNU620. We used CRISPR/Cas9 to knock out ZNF793 in the ZNF793-expressing GC cell lines SNU719 and AGS and verified the results via RT‒PCR and Western blotting (Fig. 2d & e). Compared with control cells, knockout cells exhibited significant reductions in Transwell migration and invasion. Restoring the expression of ZNF793 mitigated the effects of ZNF793 knockout in the Transwell migration and invasion assays (Fig. 3a & b). In the cell proliferation assay, ZNF793 knockout significantly reduced the proliferation rate of SNU719 and AGS cells (Fig. 3c). However, restoring ZNF793 expression resulted in increased proliferation in SNU719 and AGS cells (Fig. 3c). To evaluate the impact of ZNF793 loss on cellular apoptosis, an apoptosis assay was conducted using a dead cell apoptosis kit with Annexin V and flow cytometry-based determination of the sub-G1 fraction in SNU719 and AGS cells, both with and without ZNF793 knockout. The results indicated that there were more apoptotic cells in the ZNF793 knockout group than in the group without ZNF793 knockout (Fig. 4a-d). Compared to sgZNF793 treatment plus control treatment, sgZNF793 plus restoration of ZNF793 expression led to suppression of apoptosis in both SNU719 and AGS cells (Fig. 4a-d).

### Knocking out ZNF793 decreases the stemness of tumor cells

**Fig. 5:**
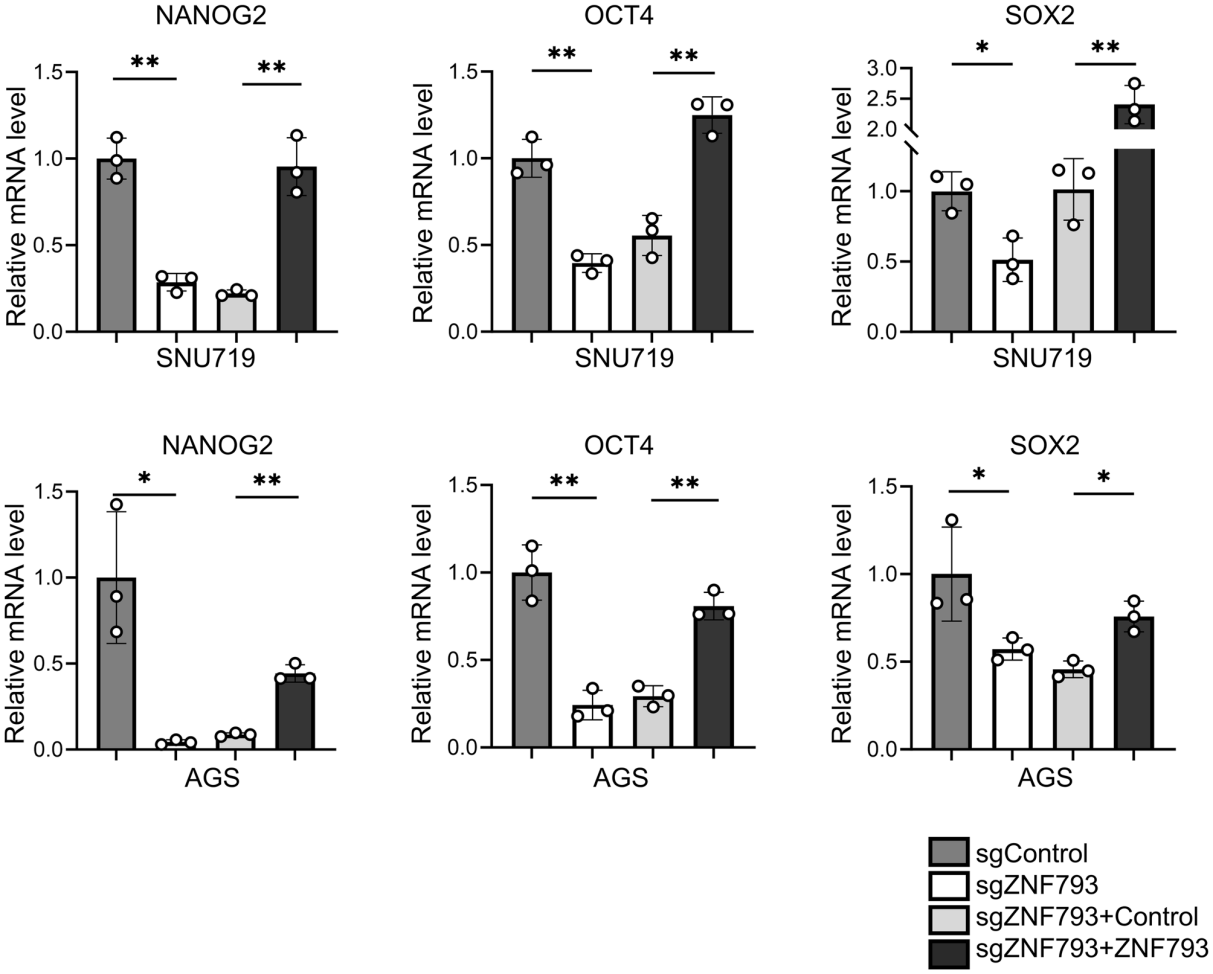
Reverse transcription quantitative polymerase chain reaction of stemness factors (NANOG2, OCT4, and SOX2) in SNU719 and AGS cells with ZNF793 knockout and restoration of ZNF793 expression

**Fig. 6:**
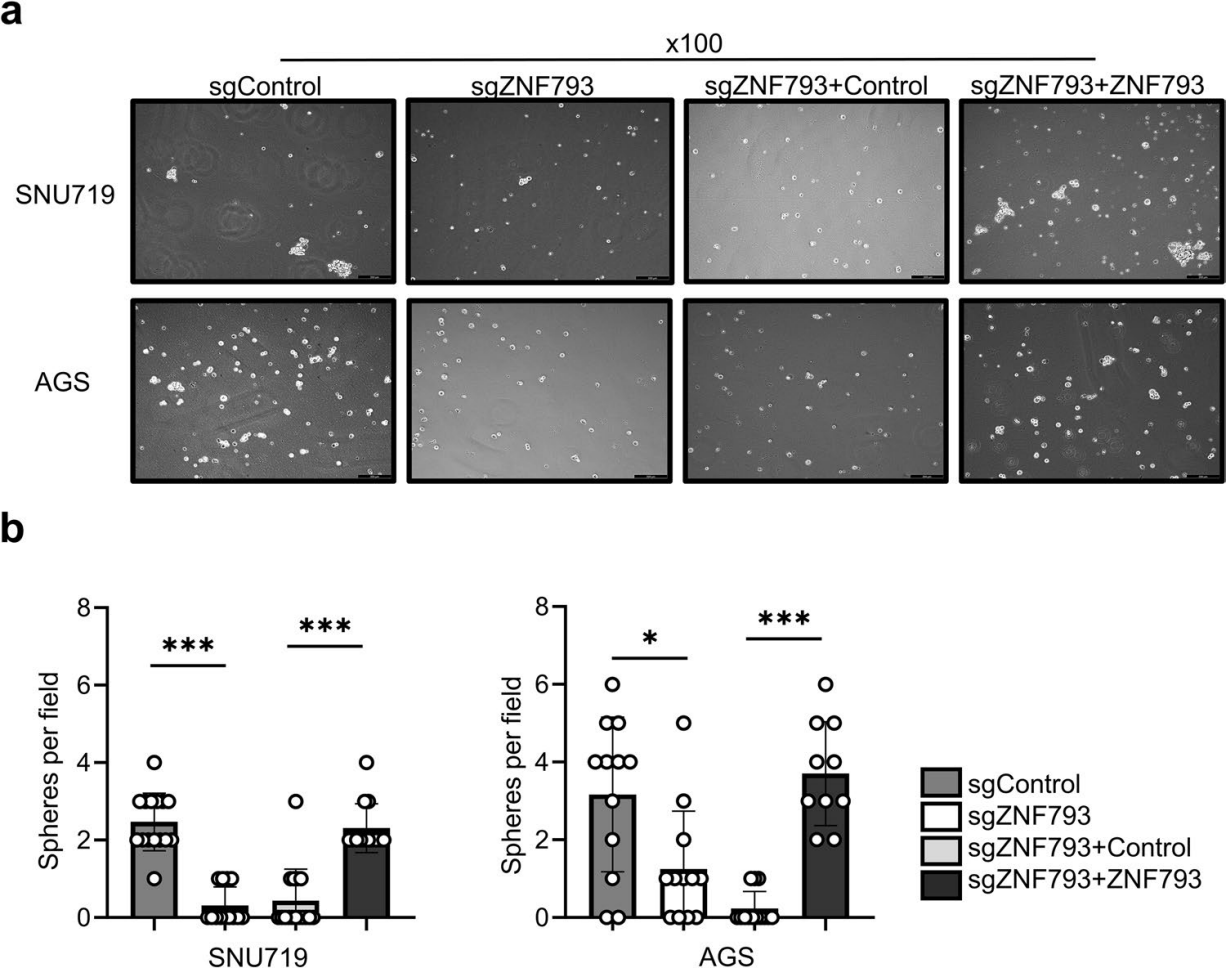
**a** Representative images of tumor sphere formation assays in cells (SNU719 and AGS) with sgNC, sgZNF793, sgZNF793 plus the control vector, or sgZNF793 plus the ZNF793 expression vector (scale bar: 200 μm). **b** Tumor sphere numbers are presented as the means ± SDs from 3 replicate wells. *P < 0.05, ***P < 0.001

**Fig. 7:**
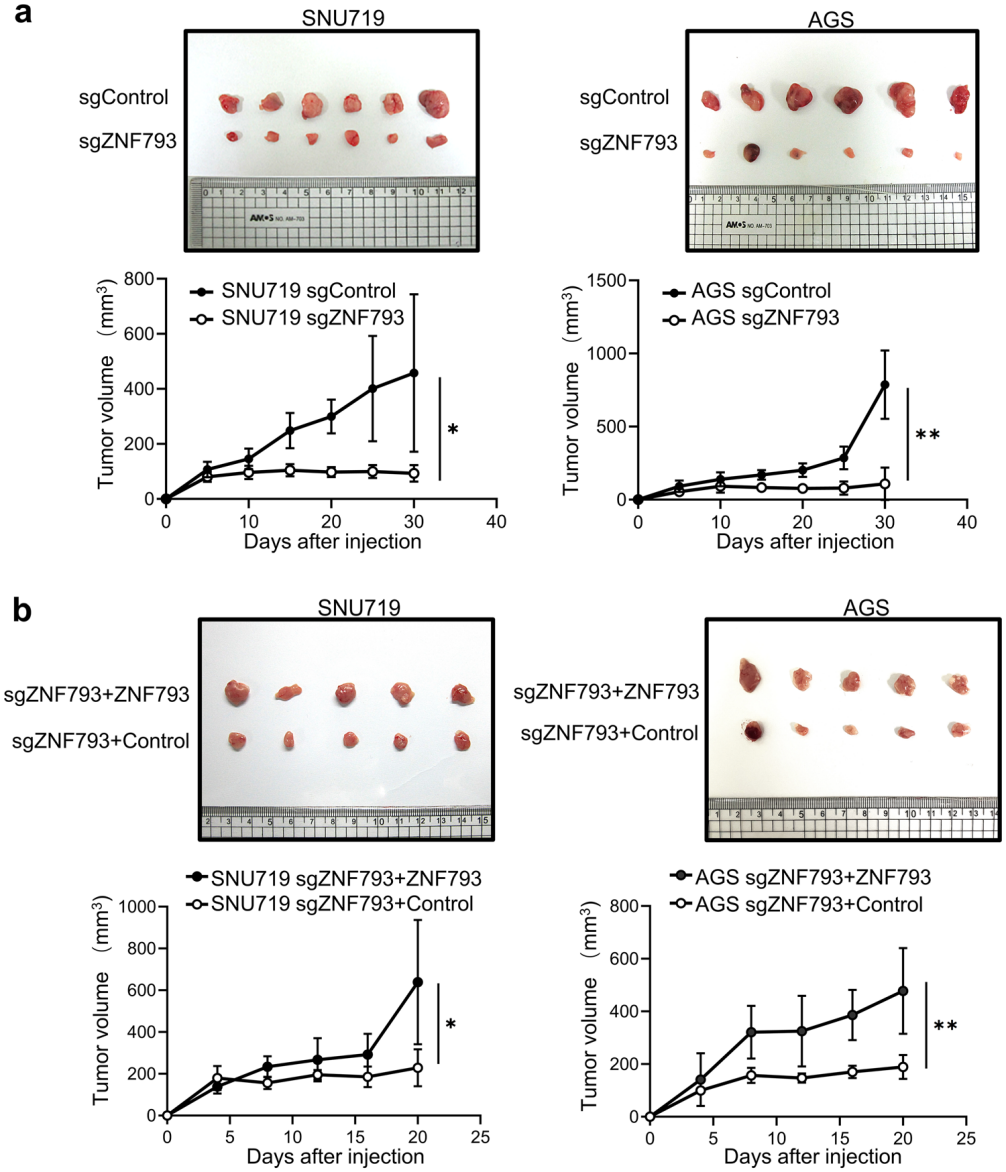
Time-dependent tumor volume (mm^3^) in xenograft mice. **a** Subcutaneous tumor growth in mouse xenografts for 30 days after injection of cells transduced with sgNC (control) or sgZNF793 (knockout). The tumor volume was measured at different time points. *P < 0.05, **P < 0.01 **b** Subcutaneous tumor growth in mouse xenografts 20 days after the injection of knockout cells with restoration of ZNF793 expression or expressing the control vector. *P < 0.05, **P < 0.01

RT‒PCR revealed that the expression of the stemness factors NANOG2, OCT4, and SOX2 was significantly reduced in ZNF793 knockout cells (Fig. 5), and these effects were reversed by ZNF793 reintroduction. In SNU620 cells, siRNA-mediated knockdown of ZNF793 also led to decreased expression of NANOG2, OCT4, and SOX2 (Supplementary Fig. 4a-c). A tumor sphere formation assay revealed that the number of tumor spheres was significantly lower in the knockout SNU719 and AGS cells than in the corresponding non-knockout cells. When *ZNF793* was reintroduced into the knockout cells, the number of tumor spheres increased (Fig. 6a & b). Similarly, ZNF793 knockdown in SNU620 cells resulted in decreased tumor sphere formation (Supplementary Fig. 4d). In the xenograft tumor assay, engraftment of both SNU719 and AGS cells with ZNF793 knockout resulted in a significant decrease in tumor volume (Fig. 7a), and restoration of ZNF793 expression resulted in restoration of tumor volume (Fig. 7b).

## Discussion

The human genome contains over 380 KZFP genes, and the well-known functions of KZFP genes in normal cells involve the imprinting of genes and the silencing of transposable elements [9, 11, 20]. The present study, for the first time, characterized the biological processes associated with *ZNF793*, including tumor stemness, cell proliferation, migration, invasion, and inhibition of apoptosis. Among the KZFP genes whose expression was altered in cancer, approximately 70% tended to be downregulated, whereas the remaining KZFP genes were generally overexpressed in most of the tumors examined compared with normal tissues [21, 22]. TCGA data revealed that the ZNF793 expression level was significantly greater in the EBV GC subtype than in other GC molecular subtypes and nonneoplastic gastric mucosal tissues (Supplementary Fig. 5). Copy number gain or gene amplification was not specifically increased in EBV GCs compared with other molecular subtypes and was not correlated with the mRNA expression of *ZNF793* (Supplementary Fig. 6a & b). The increased frequency of *ZNF793* methylation in the MSI or CIN subtypes might contribute to the decreased expression of *ZNF793*. The reason that the mRNA expression level of *ZNF793* is greater in EBV GC tissues than in nonneoplastic gastric mucosal tissues is unclear. However, considering that adjacent nonneoplastic mucosal tissues tend to exhibit intestinal metaplasia, promoter CpG island hypermethylation of *ZNF793* is likely to occur at a high frequency (Supplementary Fig. 7), resulting in decreased levels of *ZNF793* mRNA expression in adjacent nonneoplastic mucosal tissues of GC patients. 

Since EBV GC was demonstrated to harbor extreme methylation in genome-wide promoter CpG island loci [5, 23], the Kaneda team [6, 24] and the Yun team [25] have investigated the causal relationship between EBV infection and genome-wide hypermethylation by transfecting recombinant EBV into neoplastic or nonneoplastic gastric epithelial cells and comparing genome-wide methylation profiles before and after infection. Upon infection with recombinant EBV, the genomic DNA of gastric epithelial cells undergo a “methylation wave” caused by EBV infection; in this process, DNA methylation occurs on 1,662 genes within 4 weeks after infection of GES cells with recombinant EBV [24]. Methylation of the genomic DNA involves both methylated and nonmethylated promoter CpG island loci in the other molecular subtypes. Nevertheless, a small subset of genes, including *MLH1* and *ZNF793*, showed resistance to the extreme methylation wave caused by EBV infection[6]. The ability of *ZNF793* to evade DNA methylation influenced by EBV infection may be associated with its role in tumor cell stemness, which was demonstrated in the present study by the observed decrease in and reversal of intracellular stem cell marker expression, tumor sphere formation, and xenograft tumor growth upon knockout and subsequent reintroduction of *ZNF793*, respectively. It is plausible that EBV GC cells that undergo DNA methylation of *ZNF793*, which leads to inactivation of *ZNF793*, may be pro-apoptotic or outcompeted in survival by EBV GC cells that express *ZNF793*, resulting in their eventual disappearance.

Although *MLH1* methylation is observed in approximately 80% of MSI GCs, *MLH1* methylation is not observed in EBV GCs [2, 6]. Both *MLH1* methylation and mismatch repair gene mutations are not detected in EBV GCs. To elucidate the reason for the exclusiveness between EBV GCs and MSI GCs, Kim et al. [7] knocked down *MLH1* in SNU719 and NCC24 cells and reported that MLH1 knockdown did not affect cell proliferation, migration, or invasion but reduced tumor stemness, as evidenced by a decrease in CD44 expression status, tumor sphere formation, and xenograft tumor growth. In the present study, ZNF793 knockout also suppressed the stemness features of SNU719 cells. The common role of ZNF793 and MLH1 in EBV GC is tumor stemness, so it is presumed that the main reason why DNA methylation of *MLH1* and *ZNF793* is not observed in EBV GC is attributed to tumor stemness.

It is challenging to determine the reason *ZNF793* is methylated and inactivated in the EBV GC cell lines, NCC24 and YCCEL1. If ZNF793 is crucial for EBV GC survival, why is it methylated and inactivated in NCC24 and YCCEL1 cells? Because genetic and epigenetic changes occur during the establishment of cancer cell lines from cancer tissues [26, 27], *ZNF793* is thought to be epigenetically altered during the establishment of cell lines, indicating that NCC24 and YCCEL1 cells do not require the expression of ZNF793 for their establishment as cell lines. However, the near absence of methylation in EBV GC tissues might suggest that, in vivo, the expression of ZNF793 is necessary for the survival of EBV GC cells.

In conclusion, this study of *ZNF793* in EBV and non-EBV GCs provides significant insights into its role as a stemness factor. ZNF793, which is typically unmethylated in EBV GC, is crucial for maintaining cell proliferation, cell migration, cell invasion, and tumor growth in GCs with expression of *ZNF793*. Knockout experiments demonstrated that the absence of ZNF793 significantly suppressed these stemness- and oncogenesis-related processes, whereas its reintroduction restored these processes. The ability of *ZNF793* to evade methylation in EBV GC, but not in other GC subtypes, highlights its potential role in this cancer type. However, the oncogenic role of ZNF793 is not limited to EBV GC, but rather is associated with *ZNF793*-hypomethylated GC, regardless of molecular subtype. This research underscores the importance of ZNF793 as a stemness factor and provides new ideas for targeted therapies.

## Supplementary Information

Below is the link to the electronic supplementary material.

Supplementary Fig. 1a Volcano plot (right) and heatmap (left) of genes that are differentially expressed between EBV gastric cancers and MSI gastric cancers (from TCGA), generated via Sangerbox. The gene expression matrix and molecular subtype information for the TCGA gastric cancer cohort were obtained from UCSC Xena and cBioPortal, b Methylation levels of CpG sites located in the ZNF793 promoter CpG island locus according to the molecular subtypes of gastric cancer (from the TCGA). DNA methylation information and molecular subtype data for the TCGA gastric cancer cohort were obtained from UCSC Xena and cBioPortal. Compared with the other molecular subtypes, including CIN (n = 128), GS (n = 54), and MSI (n = 59) subtypes, the EBV subtype (n=24) has lower beta values for the CpG sites, and the MSI subtype has the highest beta values. CIN, chromosomal instability; GS, genome stablility; MSI, microsatellite instability. ***, P < 0.001, c Comparison of ZNF793 mRNA levels according to the molecular subtypes of gastric cancer (from TCGA). A gene expression matrix and molecular subtype information for the TCGA gastric cancer cohort were obtained from cBioPortal. ZNF793 mRNA expression levels were significantly higher in the EBV subtype (n = 23) compared to CIN (n = 105), GS (n = 49), and MSI (n = 45) subtypes. CIN, chromosomal instability; GS, genome stablility; MSI, microsatellite instability. *, P < 0.05; **, P < 0.01; ****, P < 0.0001. Supplementary file1 (TIF 9476 KB)

Supplementary Fig. 2 Correlation between ZNF793 mRNA expression and DNA methylation levels at individual CpG sites within the ZNF793 promoter region in gastric cancer samples from The Cancer Genome Atlas (TCGA). DNA methylation information and gene expression matrix for the TCGA gastric cancer cohort were obtained from UCSC Xena and molecular subtype information from cBioPortal. Each dot represents a single tumor sample. Probe IDs of Illumina HumanMethylation450 array for each site are indicated. Pearson correlation coefficients (R) and P values are shown. Most CpG sites (13 of 15 sites) exhibit significant inverse correlations with ZNF793 expression. EBV GC is highlighted in red but linear regression line represents all molecular subtypes. Supplementary file2 (TIF 26392 KB)

Supplementary Fig. 3 CpG methylation heatmap of the ZNF793 gene across TCGA gastric cancer molecular subtypes. Median beta values of 15 CpG probes mapped to different genic regions (TSS1500, TSS200, 5'UTR, gene body, and 3'UTR) were visualized by subtypes (EBV, GS, CIN, and MSI), with color-coded values indicating relative methylation levels (yellow: hypermethylation; purple: hypomethylation). Notably, the EBV subtype consistently exhibits low methylation levels at CpG sites around the transcription start site (TSS1500, TSS200, and 5’UTR), while the MSI and CIN subtypes display higher methylation levels in the same regions. Supplementary file3 (TIF 6952 KB)

Supplementary Fig. 4a Efficient knockdown of ZNF793 mRNA in SNU620 gastric cancer cells. Reverse transcription-quantitative polymerase chain reaction (RT-qPCR) showed a significant reduction in ZNF793 mRNA levels in SNU620 cells transfected with siZNF793 compared to cells transfected with control siRNA (siControl). GAPDH was used for normalization. **, P < 0.01. b Western blot analysis confirmed the decreased expression of ZNF793 protein following siZNF793 transfection. β-actin was used as a loading control. The ZNF793/β-actin densitometric ratios were calculated and indicated below the corresponding bands. c RT-qPCR analysis of stemness-associated factors (NANOG, OCT4, and SOX2) in SNU620 cells transfected with siControl or siZNF793. *, P < 0.05; ns, not significant. d Representative images of tumor-sphere formation assay in SNU620 cell with siControl or siZNF793 transfection (scale bar: 200 μm). *, P < 0.05. Supplementary file4 (TIF 12938 KB)

Supplementary Fig. 5 Analysis of ZNF793 mRNA expression levels according to gastric cancer molecular subtype (from the TCGA). Gene expression matrix and molecular subtype information for the TCGA gastric cancer cohort were obtained from UCSC Xena and cBioPortal. Expression was significantly altered among the groups: normal tissue (n = 31), CIN (chromosomal instability, n = 112), GS (genome stability, n = 48), MSI (microsatellite instability, n = 44), and EBV (n = 22). *, P < 0.05; ***, P < 0.001. Supplementary file5 (TIF 2675 KB)

Supplementary Fig. 6a ZNF793 DNA mutation types and mRNA expression levels in gastric cancer molecular subtypes (based on TCGA data) were analyzed via cBioPortal. The figure represents data for 265 samples for which both mutation profiles and expression levels were available (axes). b ZNF793 copy number alterations and mRNA expression levels in gastric cancer (based on TCGA data) were analyzed via cBioPortal. The figure represents data for 263 samples for which both copy number alteration and expression level data were available (axes). GISTIC, Genomic identification of significant targets in cancer. Supplementary file6 (TIF 5897 KB)

Supplementary Fig. 7 Methylation levels of ZNF793 across gastric lesions in multiple stages, including chronic gastritis (n=22), intestinal metaplasia (n=18), gastric adenoma (n=19), and gastric adenocarcinoma (n=36) samples. ns, not significant; ***, P < 0.001. Supplementary file7 (TIF 2823 KB)

Supplementary file8 (DOCX 18 KB)

## Data Availability

The TCGA data analyzed to generate Supplementary Fig. 1a-c and Supplementary Fig. 2, including differentially expressed genes, ZNF793 DNA methylation levels, and ZNF793 mRNA expression levels, were obtained from the UCSC Xena Browser at https://xena.ucsc.edu/ and cBioPortal https://www.cbioportal.org/. These datasets were further processed and analyzed via GraphPad Prism software. The volcano plot presented in Supplementary Fig. 1a was generated via Sangerbox, an online platform for clinical bioinformatics analysis [19].
