# Supplementary figures and images for: The oncogenic role of hypomethylated ZNF793 in gastric carcinoma: a focus on cell survival and stemness
Source: Gastric Cancer. 2025 Jun 22;28(5):814–24. doi: 10.1007/s10120-025-01632-8 (PMC12378280; doi:10.1007/s10120-025-01632-8)

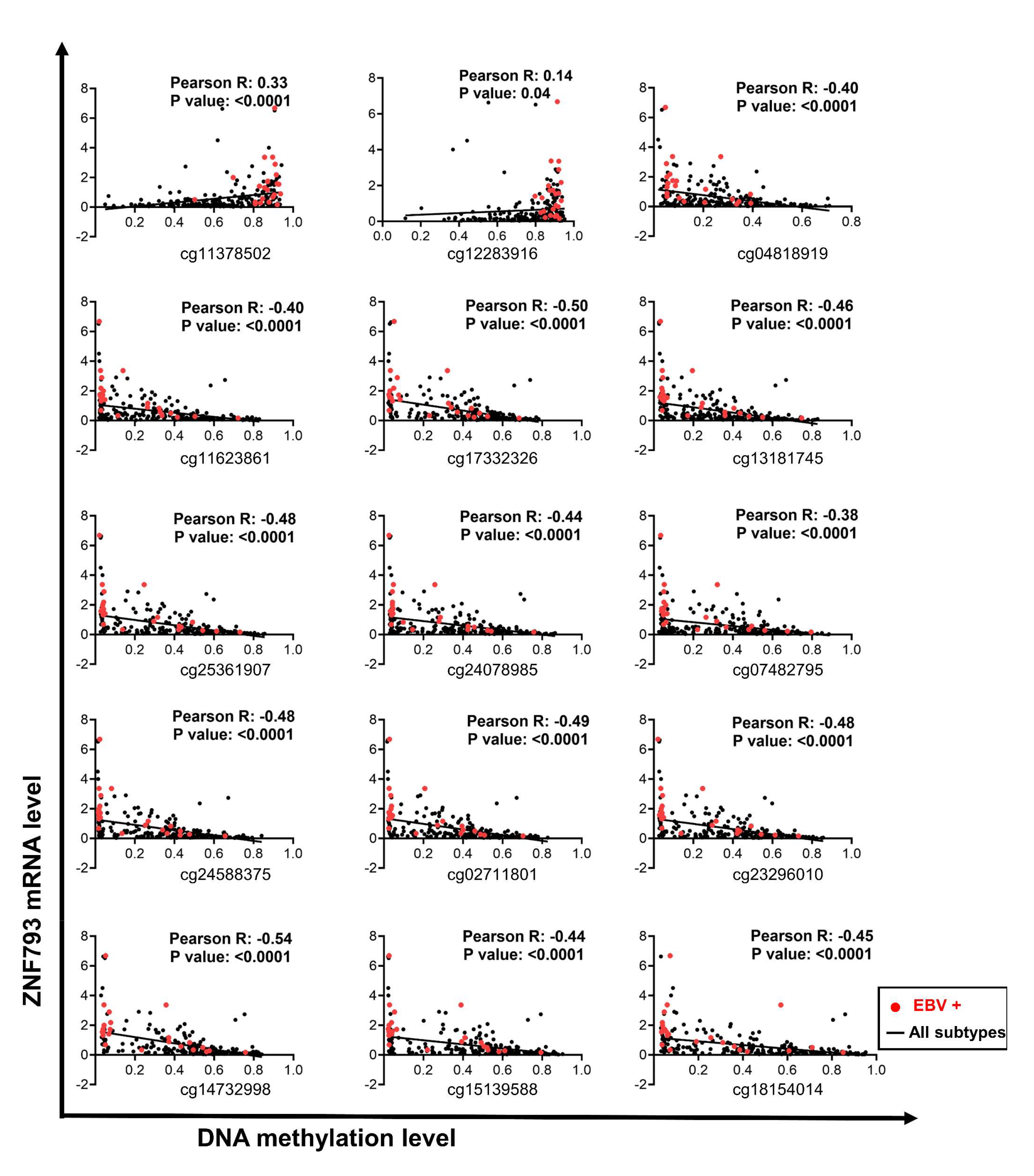

Supplement: Supplementary file 2 — Supplementary Fig. 2 Correlation between ZNF793 mRNA expression and DNA methylation levels at individual CpG sites within the ZNF793 promoter region in gastric cancer samples from The Cancer Genome Atlas (TCGA). DNA methylation information and gene expression matrix for the TCGA gastric cancer cohort were obtained from UCSC Xena and molecular subtype information from cBioPortal. Each dot represents a single tumor sample. Probe IDs of Illumina HumanMethylation450 array for each site are indicated. Pearson correlation coefficients (R) and P values are shown. Most CpG sites (13 of 15 sites) exhibit significant inverse correlations with ZNF793 expression. EBV GC is highlighted in red but linear regression line represents all molecular subtypes. Supplementary file2 (TIF 26392 KB) [file 10120_2025_1632_MOESM2_ESM.tif]
