# Supplementary material for: The oncogenic role of hypomethylated ZNF793 in gastric carcinoma: a focus on cell survival and stemness
Source: Gastric Cancer. 2025 Jun 22;28(5):814–24. doi: 10.1007/s10120-025-01632-8 (PMC12378280; doi:10.1007/s10120-025-01632-8)
Supplement: Supplementary file 8 — Supplementary file8 (DOCX 18 KB) [file 10120_2025_1632_MOESM8_ESM.docx]

Supplementary Table 1. Sequences used for the MethyLight assay

| Target | Type | Sequence |
| --- | --- | --- |
| ZNF793 | Probe | TCG CAA TTA AAT CTA CGC ACC GAC CAT TCA |
| ZNF793 | Forward primer | AAT TAC CGA ATA ACG ACT AAC AAC CTC |
| ZNF793 | Reverse primer | CGT TTT ATC GCG GTA TAA GGT CG |

Supplementary Table 2. Sequences used for sgRNAs

| Name | Sequence |
| --- | --- |
| sgControl | TAC AAC AGC CAC AAC GTC TAT |
| sgZNF793-a | TAT ACA TAA GTG CCG GGT TC |
| sgZNF793-b | ACC AGA TGT GAT CCT CAG AC |

Supplementary Table 3. Sequences of RT–PCR primers used in this study

| Gene name | Forward sequence (5’-3’) | Reverse sequence (3’-5’) |
| --- | --- | --- |
| GAPDH | GGC GTC TTC ACC ACC ATG GAG | GCC AGG GGT GCT AAG CAG TTG GTG |
| NANOG | GCC ACT ACT GTG CCT TTG AGTC | AAA GCG GCA GAT GGT CGT TTGG |
| OCT4 | CTC CAA CAT CCT GAA CCT CAGC | CGT CAC ACC ATT GCT ATT CTT CG |
| SOX2 | GCT ACA GCA TGA TGC AGG ACC A | TCT GCG AGC TGG TCA TGG AGTT |
| ZNF793 (4-6) ^a^ | GCC CTG CAG CTA AGT GAT CT | GAA GCC CAC AAC CAC ATC TT |
| ZNF793 (6-7) ^a^ | AAG ATG TGG TTG TGG GCT TC | TCC TGC TCC AGT CTG AGG AT |
| ZNF793 (7-8) ^a^ | GAG CCC TAG TAA CTG GAA CC | CTC CAT TAG GTC GTC TAC CA |
| ZNF793 ^b^ | GGA AGA AGC ACC ATG GAT TG | TCT TTC CAC AAG GGT TCC AG |

^a^ Sequences RT‒PCR primers used in Figure 2B amplify specific regions of the ZNF793 gene: ZNF793 (4-6), (6-7), and (7-8) span exons 4 to 6, exons 6 to 7, and exons 7 to 8, respectively.

^b^ RT‒PCR primers were designed to amplify specific regions of ZNF793 mRNA following sgRNA-mediated knockout.

Supplementary Table 4. Antibody list

| Antibody | Vendor | Cat. no. | Dilution for Wb |
| --- | --- | --- | --- |
| ZNF793 | Invitrogen | PA5-69001 | 1:1000 |
| α-Tubulin | Santa Cruz | sc-8035 | 1:1000 |
| β-Actin | Santa Cruz | sc-47778 | 1:1000 |
